# Assigned nurses and a professional relationship: a qualitative study of COPD patients’ perspective on a new palliative outpatient structure named CAPTAIN

**DOI:** 10.1186/s12904-019-0410-0

**Published:** 2019-03-02

**Authors:** D. G. Bove, M. O. Jellington, M. Lavesen, K. Marså, S. F. Herling

**Affiliations:** 1 Emergency Department, Copenhagen University Hospital, Nordsjælland, Dyrehavevej 29, 3400 Hillerød, Denmark; 2 Department of Pulmonary & Infectious Diseases, Copenhagen University Hospital, Nordsjælland, Dyrehavevej 29, 3400 Hillerød, Denmark; 3 Department of Palliative Medicine, Copenhagen University Hospital, Herlev and Gentofte Hospital, Herlev Ringvej 75, 2730 Herlev, Denmark; 4 Neuroscience Centre, Copenhagen University Hospital, Rigshospitalet, Section 2091, Inge Lehmanns Vej 7, 2100 Copenhagen Ø, Denmark

**Keywords:** Nurse/physician collaboration, Interpretive description, Palliative care organization, Non-malign palliative care, Qualitative research

## Abstract

**Background:**

Little is known of how to organize non-malign palliative care, and existing knowledge show that patients with COPD live with unmet palliative needs and low quality of life. With the intent to improve palliative care for patients with COPD, we changed the structure of our outpatient clinic from routine visits by a pulmonary specialist to a structure where each patient was assigned a nurse, offered annual advance care planning dialogues, and ad hoc pulmonary specialist visits. The aim of this study was to explore COPD patients’ experiences with a new and altered palliative organization.

**Methods:**

The design was interpretive description as described by Thorne. We conducted ten semi-structured interviews with patients with severe COPD from January 2017 to December 2017.

**Results:**

Patients described how the professional relationship and the availability of their nurse was considered as the most important and positive change. It made the patients feel safe, in control, and subsequently influenced their ability to self-manage their life and prevent being hospitalized. The patients did not emphasize the advanced care planning dialogues as something special or troublesome.

**Conclusion:**

We showed that it is relevant and meaningful to establish a structure that supports professional relationships between patient, nurse and physician based on patients needs. The new way of structuring the outpatient care was highly appreciated by COPD patients and made them feel safe which brought confidence in self-management abilities.

**Electronic supplementary material:**

The online version of this article (10.1186/s12904-019-0410-0) contains supplementary material, which is available to authorized users.

## Background

Chronic obstructive pulmonary disease (COPD) is a progressive disease with a significant burden for the individual and society [1]**.** Patients with COPD report a median of 11–14 symptoms which is comparable with the burden of patients with advanced lung cancer [2, 3], and several studies describe how patients with COPD live their last year of life with unmet palliative needs and subsequently low quality of life [4–9].

Danish patients with COPD clearly articulate a wish to avoid hospitalizations and readmissions and to die at home [10] . Despite this, patients with COPD are extensively present in both emergency and pulmonary departments [11], and 67% of patients with COPD die in hospitals [12, 13]. In Denmark, COPD is listed as the most frequent reason for admittance to medical departments [14].

In the last 5–10 years there has been an increasing awareness of the palliative needs for patients with COPD, and existing international guidelines recommend that patients are offered early and integrated palliative care including advanced care planning (ACP) [15–18]. Palliative care has traditionally been offered to patients within the cancer paradigm, and only limited knowledge and experience exist within the field of palliative care to patients with non-malignant diseases [19]. Several studies recommend that patients with non-malign lung disease do not receive the same standards of palliative cares as patients with cancer, instead new or altered organizations must be developed and evaluated to improve palliative care for patients with COPD and other non-malign disease [4, 20, 21]. In this project palliative care is defined as an approach that improves the quality of life of patients and their families facing the problem associated with life-threatening illness, through the prevention and relief of suffering by means of early identification and impeccable assessment and treatment of pain and other problems, physical, psychosocial and spiritual (WHO).

To comply with existing recommendations [15], we changed the structure of the outpatient clinic from a traditional structure with routine control visits by a pulmonary specialist toward a more patient-centered structure. The new structure was named CAPTAIN; an acronym for Comprehensive And Prospective Treatment and Individual Nursing and was based on altering existing roles and tasks between nurses and physicians. CAPTAIN was developed and implemented within the deparment’s existing budget and not funded by any research grant. The key elements in CAPTAIN were that each patient was assigned a CAPTAIN-nurse (c-nurse) and offered annual ACP dialogues [5, 22]. The c-nurse had overall responsibility for establishing and maintaining a professional relationship with the patient. The patients could contact the c-nurses every workday between 8 and 10 am by phone. Approximately 600 patients were affliated CAPTAIN. A c-nurse had an average of 85–100 patients. The frequency and type of contacts depended on individual needs of patients and varied over time. The organization of CAPTAIN is described in a previous paper [23], and the health professionals’ expectation and experiences with the new structure in another paper [24].

CAPTAIN was initiated with the aim of improving palliative care for patients with severe COPD and thereby increase their quality of life. However, we do not know if this re-organization and similar organizations is perceived as being of value to patients.

## Methods

### Aim

The aim of this study was to explore COPD patients’ experiences with the new palliative organization named CAPTAIN.

### Design

The methodology was interpretive description ad modum Thorne [25] and based on semi-structured interviews with 10 patients with severe COPD. Relatives were present during the interview in two cases. The relatives’ experiences were not explored specifically, but contributed with nuancing the patient perspective.

### Setting and participants

The sampling strategy was targeted purpose-oriented [25], and based on the participants being reprensentative for the target population. We sought variation in gender, age, FEV_1,_ oxygen supply, and social status. Participants were recruited from a pulmonary outpatient clinic located in the Capital Region of Denmark, Copenhagen. All were diagnosed with severe COPD [15] and had been affiliated CAPTAIN for at least one year. A total of 13 patients were invited to participate, three declined due to fatigue or hospital admission. Characteristics of the participants are presented in Table 1.

**Table 1:** Characteristics of participants (*n* = 10)

ID	Age (year)*	Sex	FEV_1_ (%)	CAT	BMI	MRC	Oxygen	Social status
1	54–74	Female	13	21	22	5	Yes	Alone
2	54–74	Male	21	26	22	5	No	Alone
3	54–74	Male	28	26	28	5	Yes	With partner
4	54–74	Male	46	13	19	3	No	Alone
5	54–74	Female	15	22	24	5	Yes	With partner
6	54–74	Male	29	15	35	3	No	With partner
7	54–74	Female	24	26	17	5	Yes	With partner
8	54–74	Male	35	21	23	4	No	Alone
9	54–74	Female	22	38	13	5	Yes	Alone
10	54–74	Female	23	23	18	4	No	With partner

*Ages are presented as a range to ensure patient anonymity

Abbreviations**:** *ID* Identification number; *FEV*_*1*,_ Forced expiratory volume in one second percent predicted; *CAT* The COPD Assessment Test range 0–40; *BMI* Body mass index; *MRC* Medical Research Council Dyspnoea Scale range 1–5

### Data collection

All interviews, except for one, were conducted in the home of the patients from January 2017 to December 2017 and lasted 20–39 min (mean 29). The interviews were conducted by two experienced interviewers (first and second author). All interviews were recorded, and immediately after the interview, the interviewers wrote reflective notes describing their first impressions and observations. The interview technique was open-ended questions with follow-up questions related to the patient’s responses and current focus. An interview guide with opening questions was developed to support the interviewers and direct the interviews. The opening questions are shown in Additional file 1.

### Data analyses

All 10 interviews were transcribed verbatim and resulted in a total of 85 pages (Calibri, size 11). All transcripts were organized in QSR NVivo 11© software (QSR International Pty ltd, Cardigan, UK). The data collection and analysis was conducted as an iterative process and the interview-guide was continuously shortened and focused as our insight increased.

The first author read each interview and notes to obtain an overall impression. Data was coded into very broad-based categories in NVivo. These categories were continuously discussed with the last author. Next, we searched for intuitive meaning units and patterns in the participants’ experiences with CAPTAIN. The identified themes and subthemes were explored by describing the content of each theme and further explored and tested in patients’ interview and by discussion among all authors. Afterwards we constructed multiple iterations of the themes by adding new themes, re-organising themes and merging themes several times. To move from the descriptive to the interpretive phase, the authors continuously asked questions like: what am I seeing, does this surprise me and why, what does this mean for the patients and what are the consequences and recommendations for clinical practice.

Finally, the first author re-read the original transcripts and the notes to ensure consistency among the themes and the way the informants described their experiences. All steps in the research process have been conducted in collaboration between two or more of the members of the research team. Quotes illustrating the themes are presented in a concentrated and edited form to increase readability.

### Rigour

With an awareness of the limitations and ongoing debate about the appropriateness of using member check [26], we presented our preliminary themes for a group of patients with COPD and informal caregivers. As part of developing and implementing CAPTAIN a patient and informal caregiver feedback group was established consisting of five patients with severe COPD and three informal caregivers [23]. The preliminary findings were presented by the first and third author at one of their meetings. There was agreement among the group members that the themes were recognizable and made sense. The member check did not lead to any change in themes, but the group members contributed to validate the interpretive authority [25]. The COREQ-criteria for reporting qualitative research were used.

## Results

Our analysis revealed that the new organization contributed to increased quality of life for patients with COPD, although a few also described being worried and skeptical toward the new structure of outpatient care.

### A professional relationship between patient and c-nurse is the key element of CAPTAIN

All patients, without exceptions described the professional relationship with their c-nurse as the key element of CAPTAIN, and the element that made the difference for them. The fact that the patients’ life and medical history were known by the c-nurse made the patients feel unique and not just one in the crowd, which further strengthened the relationship between patient and c-nurse. This feeling was highly valued by the patients and had a major impact on the feeling of being safe and subsequent quality of life. The words security and feeling safe was repeatedly used by the patients in the description of their experience with CAPTAIN.

*• In this health system, you constantly meet new people, people you do not know, every time you go through something. So, it’s definitely getting to know each other that is of great value. She knows my background and what other issues I’m dealing with. It is also important because you are so psychologically affected when you are ill (ID 7).*

The professional relationship with the c-physician was also portrayed as important, although the patients’ differed in the description of how well they knew their c-physician. The patients perceived the c-nurse and c-physician as a team, and were aware that the c-nurse was supported by a c-physician. The c-nurse was the one who initiated and maintained the professional relationship with the patient, and because the relationship was characterized by continuity and stability, the patients specified that it did not mean as much if it was not the same physician they met each time they came in for a consultation.

The patients did not emphasize the annual ACP as something special or troublesome. The relationship and trust in the c-nurse and c-physician meant that issues related to future treatment and end of life decisions felt natural to discuss. None of the patients had experience the ACP dialogue as unsettling, and several of the patients had difficulty separating ACP from other dialogues they had with their c-physician or c-nurse.

A drawback of the close professional relationship with the c-nurse was described by a patient who experienced the retirement of her c-nurse. The patient described a feeling of being detached, abandoned, and left out. The patient was not able to enter a new professional relationship with another c-nurse for several months, and described herself as being somewhat hostile toward the new c-nurse.

*• My old c-nurse, whom I have known for a long time has retired. I thought; No ... you cannot let me down. We were so good together and I do not want a new c-nurse. Somewhere, I was almost hostile to my new c-nurse. I thought, quite honestly, now nobody’s talking to me anymore, and of course there was. It is also your own responsibility - it also depends on yourself, the signals you send, and I think I was very resistant because I would really have liked to hold on to my old c-nurse. I know it is nonsense (ID 10).*

This patient described how the feeling of being safe was threatened by the fear of detachment.

The professional relationship between patient and c-nurse did not develop by itself and required that patients experienced their c-nurse as competent. A competent c-nurse was described by the patients as a nurse who was experienced in nursing, had biomedical expertise, instrumental skills, and expert knowledge about all aspect of the COPD disease. These competences did not in itself constitute a c-nurse in the perspective of the patients. The patients described how it was imperative that the c-nurse could balance the combination between being biomedical oriented and at the same time holistic in her approach. It was this act of balance that established the c-nurse as competent in the view of the patients and that paved the way for ensuring trust and confidence in the professional relationship between patient and c-nurse.

*• I call her and ask different questions about oxygen, medicine, and if I cough or if I’m miserable with myself. No matter what, my c-nurse and I can talk about it without me having to leave the house, which is important (ID 2).*

Several patients described how they through years of experience with the healthcare system countless times had met both nurses and physicians, who did not master a biomedical and a holistic approach at the same time and how this lack of blending skills diminished the quality of the relationship between patient and healthcare professionals.

### Continuity increased the quality of treatment and care as well as strengthened the patient self-management abilities

The patients explained how they experienced a higher quality and satisfaction with the treatment and care offered in the CAPTAIN-structure compared with before CAPTAIN and other traditional structures. Because their c-nurse and c-physician knew them and their course of disease, patients could move directly to talking about for them import issues. Whereas in the traditional structure they normally spend quite a lot of time updating their health professionals on their disease leaving them with only little or no time to talk about what they considered important in their everyday life.

*• It makes me feel safe that I do not have to tell my story over and over again. Before CAPTAIN, when I came every three months, I was always met by a new nurse and a new physician. I had never seen the doctor before, and experienced several episodes where I did not understand what the doctor said. During the consultations, the physician was occupied reading my journal and just looked at the results of my spirometry or blood samples and then he said; Well, see you again in half a year. Those visits were no help for me (ID 1).*

Another derived consequence of the CAPTAIN structure was the possibility for patients to set the agenda when meeting with their c-nurse or c-physician, and how this influenced their involvement in treatment and care and thereby strengthened their self-management abilities.

Several of the patients expressed how their c-nurse and c-physician had prepared them for a worse turn of their disease, and to respond promptly to symptoms of exacerbations. Several of the patients had been handed sputum sets and acute medicine, and after telephone contact with their c-nurse they could immediately initiate their self-treatment plan based on advice or recommendations from their c-nurse and c-physician. Patients experienced an increased sense of control but differed in the way the feeling was achieved. Some patient felt in control through self-management plans, others through an awareness and trust that necessary care from the c-nurses and c-physicians in case of disease deterioration was avaible.

### Availability of the c-nurse reduces number and lengths of hospitals admissions

All patients had experiences with phoning the c-nurses, and they described this possibility as being of major importance for their feeling of safety and something that had prevented their symptoms from getting worse and prevented hospitalization. The professional relationship with the c-nurses eliminated any potential barriers for contact, and the c-nurses could help the patients per telephone because of the already established relationship. The fact that the CAPTAIN-phone was answered by different c-nurses did not reduce the patients’ feeling of security or receiving adequate help.

*• It meant I avoided being hospitalized and I do not want to be hospitalized. If I had gone to my GP, then I would have been admitted to the hospital. Right away. I have tried it so many times before and I do not think it is fun. Firstly, there is a lot of noise and turmoil in the hospital and, secondly, it is much better to be home in your own bed in peace and quiet. As long as you have someone you can contact or who can care for you. So, in that way, CAPTAIN works perfectly (ID 4).*

The patients experienced that they were earlier diagnosed and treated compared to before CAPTAIN or if they had contacted their General Practioner (GP). The majority of the patients chose to contact their c-nurse instead of the GP and described several reasons for this disposition. Transporting themselves to their GP was perceived as a major barrier; secondly it was difficult for the patients even to get through to the GP on the telephone and they experienced that the GP had limited knowledge about COPD. In several cases, the patients did not give their GP an opportunity to interfere because it was easier for them to contact their c-nurse and they had the feeling of being relevantly helped through the CAPTAIN organization.

*• Because I can get advice and guidance and the c-nurses and c-physician have been so much at the forefront of things, I have antibiotics at home in case of an infection. I just send a saliva test and then I can actually start taking the medicine as soon as I have the result, from my c-nurse. I do not have to transport me myself to my GP and explain everything to him. The c-nurse has given me clear instructions on what to do and I have the things I need at home to react on a suspicion of an infection. (ID 9).*

Several of the patients described how they during hospital admission where visited by their c-nurse or met their c-physician in the ward. These visits or meetings were significant to the patients. The visits were not described as social visits, but more like an opportunity for the patients to discuss the actual treatment with their c-nurse or c-physician which gave them a feeling of confidence, safety, and hope, for recovery. The c-nurses offered to call or visit the patients after discharge, this offer made the patients feel secure and this relieved any potential anxiety related to the hospital discharge.

### Meaningful organizational changes strengthened patient’s confidence in the health system

The patients described how they prior to CAPTAIN used to see their physician every third to sixth month for a routine control. Some of the patient experienced these visits as comforting and others considered them as a waste of time. The agenda of the visits would typically be set by the physicians and involved assessment of lung function, medical status and adjustments if necessary.

The elements of continuously contact with a c-nurse and the possibility of consultations with a c-physician in case of sub-acute illness or increasing burden of symptoms were considered relevant by patients and they fully understood and supported the underlying rationale for the altered organization. However, one patient described how she felt cheated at first. At the time of CAPTAIN implementation, the patient did not see the benefit of replacing four annual physician visits with one annual ACP and the possibility of ad hoc consultations. The recentness persisted for a long time where the patient was in a stable phase of her COPD. Her opinion changed as she later was affected by repeated episodes of deterioration and made use of the opportunity to have ad hoc consultations by a physician. This changed her attitude, and at the time of the interview she described herself as a great advocate and believer of CAPTAIN. This patient expressed that the health professionals’ resources were utilized better and given to those who needed the most.

*• I have been pleasantly surprised. At first, I thought that reducing consultations from four to once a year was a way for the health care system to save money. My first impression was: how unfair! I went every three months, and now I am only seen once a year – However that’s not the way it is because I can come when I need. It can also be stated as if there is no need; there is no reason to come (ID 10).*

The possibility to get help in case of deterioration was highly valued by patients. They described the altered organization as sensible as it used the healthcare professionals’ resources on the most needing patients. Most of the patients had several years’ experience with rigid healthcare organization that did not in any way support their needs or utilize healthcare resources optimally, in their opinions. The CAPTAIN organization had strengthened patients overall trust in the healthcare system, because it became clear to them that the organization did not waste time and money but instead sought to use existing resources in a way that benefitted the patients most.

## Discussion

The patients described very consistently that it was the professional relationship with their c-nurse they valued most and that the relationship with their c-nurse positively influenced their ability to self-manage their life. The patients differed in the way they described their c-nurses’ role. Some patients felt increasingly in control of their COPD related to their c-nurse teaching and supporting self-management strategies. Others expressed how they were convinced that in case of deterioration their c-nurse would provide necessary care. Both scenarios helped patients feel safe, in control, and reduced the feeling of being helpless. The feeling of being safe was highly valued by the patients that live with the unpredictable course of COPD [27–29] . This sense of safety and connection could vanish if the c-nurse was replaced. Because the relationship was based on a subject to subject relationship the patients experienced the thought of losing their c-nurse as worrying and could provoke anxiety. An alteration to accommodate this threatening feeling could be to make a workflow describing how a c-nurse must be replaced to make sure that all patients are helped into a relationship with the new c-nurse. Another alteration could be to affiliate patients to a c-nurse team of 2–3. But the question is if a structure of c-nurse teams could generate the same positive feelings found in this study. We would recommend further research into this team model.

It is well known that self-management support can improve health-related quality of life [30], and our findings do in accordance with the findings of Leine et al. [31] illustrate how the feeling of being safe is a central theme and somehow relates to the patients’ ability to self-manage life with COPD. For COPD, self-management to be effective, patients’ psychosocial needs, alongside medication and exacerbation management must be prioritized [32] . Anxiety is highly prevalent in COPD, with prevalence rates up to 46% in outpatients [9] . A hypothesis could be that the feeling of being safe is associated with a low level of anxiety, and that the majority of the perceived benefits and values of CAPTAIN is mediated through relief of anxiety symptoms.

Our findings show that self-management support must combine care and support regarding medication and other COPD specific interventions combined with psychosocial support. The patients described how the c-nurses must possess both skills as an experienced pulmonary nurse and at the same time be holistic and empathic in the approach. The mastery of the combination of skills is in the patients’ perspective what makes a good c-nurse and differentiates her from nurses patients previously have met. However, it is unclear whether it was the c-nurses’ pulmonary skills or whether it was the patients’ experience of being met by a well-known nurse who was willing to engage and support patients in all aspects of living with COPD, that patients valued most. The patients stated that it was important that the c-nurse was a pulmonary expert, but it was the holistic skills that the patients emphasized and the ones that were described as affecting patients’ ability to self-manage their lives with COPD the most and subsequently provide a sense of being safe and relieving anxiety.

The professional relationship with the c-nurses did also impact the perceived quality of the meetings with the c-physicians both in case of acute illness or scheduled ACP. A meeting with c-nurse and c-physician was scheduled to 20–30 min and the patients described how the continuity in the CAPTAIN structure meant that they no longer needed to use the sparse time available to update the health professionals but instead could move directly on to talking about issues that were important to them. The patients clearly experienced a quality improvement in the meetings, and we assume that the increased time and possibility to discuss health related problems with the c-physician and c-nurse could impact patients’ outcome and self-management ability in a positive way [33] . Although the scheduled time is the same, the content of the meetings had clearly changed in the new structure leaving more time available to discuss problems or issues relevant to the patients.

Several studies have shown that knowledge about COPD and proactive treatments plan contenting patients’ preferences for treatment and care can make patients feel safer and less anxious [16, 34] and thereby contribute to increase the patients’ quality of life. However, in contrast to the CAPTAIN structure, existing literature shows that ACP is surprisingly uncommon in COPD, properly due to barriers in both patients and health care professionals [34] . In our study, no barriers or ambivalence in engaging in ACP was found. The established professional relationship led to patients feeling safe and confident in their c-nurse and c-physician and so they could discuss end of life and other sensitive topics without any concerns. The patients described that they continuously discuss issues related to the level of treatment and/or end of life with their physicians and nurses. That way, one can say that all or no dialogues in the CAPTAIN structure contained elements from the ACP, but without the very schematic structure that is often described and recommended in the literature [22, 35] . In CAPTAIN, ACP is discussed when patients have a need and focus is on the content of the dialogue and not on the structure or the preparation of a written product. The schematic structure with annual or semi-annual ACP based on a conversation guide or form of checklist makes planning manageable for the organization, but it can be discussed whether it is the optimal solution for the patients. Our findings indicate that in a structure like CAPTAIN, ACP is not something fragmentized that must fit into a scheme, but a rather a natural part of COPD treatment and care.

The way the new CAPTAIN structure targeted focus on patients with the greatest need made intuitively sense for the patients. Because the patients perceived it as a responsible and appropriate way to spend existing resources, their confidence in the health professionals increased. The one skeptical patient changed her attitude as her disease worsened and the need for support arose. The patients in our study suffered all from severe COPD and they all, in accordance with other populations, described a wish to avoid hospitals admission [36], and had the experience that the new organization contributed to prevent this. It was the combination of availability and the professional relationship with the c-nurse that made it easy for the patients to react promptly on increasing symptoms or other needs of support. The patients described no barriers in contacting their c-nurse by phone, and often it was first choice opposed to contacting the GP. Transportation to the GP was difficult and the experience that an acute visit to the GP often resulted in hospitalizations was perceived by the patients as a barrier for contacting the GP.

The patients described how they felt safe contacting their c-nurse for advice when they suspected an exacerbation or they needed emotional support, and how they speculated that promote diagnostics and treatment prevented hospital admissions. “The majority of the patients were educated in self-management strategies by their c-nurse and c-physician and knew what action to take both on weekdays, in weekends, or out of hours”.Telephone consultation was a possibility because of the already established relationship between patients and c-nurse and the initial and most frequent form of contact. Home visit by the c-nurse, ad hoc outpatient visits with the c-physician, referral to the GP, or hospital admissions were next step if the problem was beyond what could be solved by advice or support over the phoneEach contact between c-nurse and patient was documented in the electronic patient report. These reports or summaries were sent to the GP when changes in the condition or treatment of the patients occurred.

One could argue that the perspective of the c-nurse and GP can be different and some options of treatment could be delayed if patients contact c-nurses instead of the their GP. The choice of whom to call are the patients and when patients call the c-nurse with a COPD related problem, the c-nurses role is to help and support within her level of competences. Often the alternative is not calling health care professionals at all with the risk of the condition deteriorating and resulting in an emergency call. The patients described how they felt safe consulting their c-nurse for advice in case they suspected an exacerbation, or they needed emotional support, and how they perceived this possibility to react early as something that potentially would prevent hospital admissions.

CAPTAIN was developed and implemented within an existing economic budget with the aim of improving treatment and care of patients with COPD [23, 24]. The CAPTAIN structure was found to be feasible and today CAPTAIN constitute standard care for all patients affiliated to the pulmonary outpatient clinic. The traditional outpatient structure that CAPTAIN replaced, can also be seen in other outpatients clinics for chronic disease like dementia, heart disease, diabetes, etc. We hope our findings can inspire others to reflect on and maybe change their normal workflow and ways of thinking from standardised outpatient visits to individualised care, based on the patient’s needs.

### Strengths and limitations

It is considered a strength that we were able to include participants who suffered from severe COPD with a high CAT, MRC, and a low FEV^1^ and thereby represent the characteristics of the CAPTAIN population. The sample size may be perceived as small, however for a qualitative study 10 participants with insight and experiences within the given field are considered quite sufficient. We considered our data to be rich and nuanced, and by the end of the last interview there was a redundancy in the data in relation to the themes identified enhancing the reliability of the analysis. Trustworthiness were established by all authors being involved in the analysis, discussing themes and subthemes in an iterative process. The author group was interdisciplinary and affiliated departments at different hospitals in the Capital Region of Denmark, reducing the risk of bias in form of preconception and prejudices influencing the analysis and interpretation.

## Conclusions

The new palliative structure CAPTAIN supported an individualized professional relationship between patient and c-nurse and this relationship positively impacted patient’s ability to self-manage their life with COPD. Patients felt safe and trusted their c-nurse and c-physician, which meant that no topic was too sensitive to discuss, including themes related to end of life. ACP was not perceived as something special or fragmented, but as an integrated part of all dialogues with the health professionals. The patients valued and described how the quality of meetings with the health professionals had improved because the patients no longer needed to start every meeting with updating the health professionals. Instead the available time could be used to discuss what constituted a problem for the patient.

## Additional file

Additional file 1: Interviewguide with opening questions. (DOCX 15 kb)

## Abbreviations

ACP: Advanced Care Planning
CAPTAIN- physician: C-physician
CAPTAIN: Comprehensive And Prospective Treatment And Individual Nursing
CAPTAIN-nurse: C-nurse
COPD: Chronic Obstructive Pulmonary Disease
ID: Interpretive Description
