# Supplementary material for: Assigned nurses and a professional relationship: a qualitative study of COPD patients’ perspective on a new palliative outpatient structure named CAPTAIN
Source: BMC Palliat Care. 2019 Mar 2;18:24. doi: 10.1186/s12904-019-0410-0 (PMC6397743; doi:10.1186/s12904-019-0410-0)
Supplement: Supplementary file 1 — Interviewguide with opening questions. (DOCX 15 kb) [file 12904_2019_410_MOESM1_ESM.docx]

**Supplementary file 1. Opening questions for the interviews**

- Please, tell me about a normal day in your life.
- What do you think about the CAPTAIN-structure?
  - Does it make sense and why?
- What are your experiences with using CAPTAIN?
  - Please describe an episode where you used CAPTAIN.
  - What worked well and what should be amended.
- How does the CAPTAIN differ from other structures?
- Please tell me about your CAPTAIN-nurse and your CAPTAIN-physician.
  - What do you think are important competences for a CAPTAIN-nurse and physician?
- Please tell me if CAPTAIN influences on your ability to master your disease?
  - Please describe why and how.
- How much do you think about the risk of worsening of your disease?
  - What do you do in case of an exacerbation?
  - What do you think about hospital admissions?
  - Do you have any thoughts about end of life?
- What is your experience with the advance care planning dialogue?
  - Did you bring any relatives?
  - Did you talk about what was important to you?
